# Using Big Data to Identify Impact of Asthma on Mortality in Patients with COVID-19

**DOI:** 10.3233/SHTI220473

**Published:** 2022-05-25

**Authors:** LYU Jinyan, CUI Wanting, FINKELSTEIN Joseph

**Affiliations:** a Icahn School of Medicine at Mount Sinai, USA

**Keywords:** COVID-19, Conditional Logistic Regression, Mortality

## Abstract

The goal of this paper was to assess if mortality in COVID-19 positive patients is affected by a history of asthma in anamnesis. A total of 48,640 COVID-19 positive patients were included in our analysis. A propensity score matching was carried out to match each asthma patient with two patients without history of chronic respiratory diseases in one stratum. Matching was based on age, comorbidity score, and gender. Conditional logistics regression was used to compute within each strata. There were 5,557 strata in this model. We included asthma, ethnicity, race, and BMI as risk factors. The results showed that the presence of asthma in anamnesis is a statistically significant protective factor from mortality in COVID-19 positive patients.

## 1. Introduction

The COVID-19 pandemic brought about 64,062,060 confirmed cases and resulted in 846,463 deaths in the United States [1]. Such a high number of positive patients significantly strained medical care delivery. One way to optimize healthcare resources is to identify potential severity of patients in advance. Patients with chronic respiratory diseases (CRD) who contracted SARS-CoV-2 may potentially suffer more severe consequences from COVID-19. A substantial proportion of CRD patients have asthma. A study from Scotland showed that adults with asthma had higher hospitalization, ICU admission, and death than those without asthma [2]. Our study was aimed at assessing whether mortality in COVID-19 positive patients is affected by a history of asthma in anamnesis. In this study, big data repository from a nation-wide collaborative was used to compare mortality rates in COVID-19 positive patients with history of asthma and COVID-19 positive patients without CRD.

## 2. Methodology

This study used the data from National COVID Cohort Collaborative (N3C) Data Enclave. N3C is a national wide COVID‑19 clinical data hub that contains demographic and clinical characteristics of patients who have been tested for or diagnosed with COVID-19. The dataset contains over 10 billion rows of data, 3.3 million COVID test-positive patients, and 9.4 million total patients [3]. Due to the requirements of HIPAA, all analyses were performed on N3C’s official data enclave website.

We included adult patients (age >= 18) who underwent COVID-19 testing between January 1, 2020 and April 30, 2021 and had valid test results. A COVID-19 test extraction was based on the validated LOINC codes. A COVID-19 test result was defined as positive if the result was reported as ‘Positive’ or ‘Detected.’ A test result was negative if the result was reported as ‘Not detected’ or ‘Negative.’ A COVID-19 negative patient was defined if all COVID-19 tests were negative. A COVID-19 positive patient was defined as having at least one positive test result. Death of patients deceased within 90 days after positive COVID-19 testing was attributed to COVID-19. The mortality rate in these patients was defined the patients’ COVID-19 mortality rate.

For each COVID-19 positive patient, analyses included gender, age, race, ethnicity, BMI, and state of residence in US. Asthma was identified by ICD-10 code J45. We included asthma patients without other CRD. Patients without CRD were defined as having no documented COPD (J42-J44), asthma (J45), and lung cancer (C34) in the condition occurrence table. The age-adjusted Charlson comorbidity index score was calculated from patients’ history using ICD-10 codes [4]. We divided age into four age groups 18– 35, 36–50, 51–75, and 76+ as age confounder. The Charlson comorbidity score was stratified into four levels (0–2, 3–4, 5–6, and 7 or higher) as a total comorbidity status. We removed patients with missing values or extreme outliers in age, gender, ethnicity, race, state, and BMI. All data was extracted from the condition occurrence table and then merged with independently identified patients in the patient table. PySpark was used to perform analyses of billions of medical records in N3C.

The analyses described in this publication were conducted with data or tools accessed through the NCATS N3C Data Enclave https://covid.cd2h.org and N3C Attribution & Publication Policy v 1.2-2020-08-25b supported by NCATS U24 TR002306. This research was possible because of the patients whose information is included within the data and the organizations (https://ncats.nih.gov/n3c/resources/data-contribution/data-transfer-agreement-signatories) and scientists who have contributed to the on-going development of this community resource [3].

### 2.1 Statistics model

We applied a random forester model to calculate feature importance. The result showed that age, total comorbidity score, and gender were the most important features 0.498, 0.300, 0.033, respectively. This result is in accordance with a recent paper analyzing CRD using a cluster analysis that showed that age, total comorbidity score, and gender were important factors [5]. We employed propensity-score matching method to reduce the effect of confounders. Age, total comorbidity score, and gender were used for matching. Using R package ‘matchit’, the nearest neighbor matching method matched two groups with a ratio of 1:2 (case: control) on age status, comorbidity status, and gender. All patients who only have asthma were assigned into the case group and matched accordingly with non-CRD patients who belonged to the control group. Then, we calculated descriptive statistics of the case and control group independently in the unmatching group. We applied conditional logistic regression in the case-control study through R package ‘survival’. Conditional logistic regression was chosen because of the imbalance between asthma patients and non-CRD patients. The statistically significant level was alpha equal to 0.05.

## 3. Result

A total of 48,640 COVID positive patients were included in this study, of which 5,557 patients were in the case group with one or more CRD, and 43,083 patients were in the control group without CRD. After we matched two groups with propensity-score matching method, there were 5,557 patients in case group and 11,114 patients in control group. Table 1 depicts the mortality rate of patients in different conditions. In the unmatched group, 1658 (3.9%) of non-CRD patients died due to COVID. The mortality rates due to COVID-19 in asthma patients were 2.9% (n=159). In the matched group, the overall mortality rate of asthma patients (2.9%) was significantly lower than that of patients without CRD (4.1%). Non-CRD patients in the matched group showed a higher mortality rate compared with non-CRD patients in unmatched group. This may be explained by bias reduction in matched cohorts by age, comorbidity score, and gender. Our analysis showed that asthma patients exhibited lower mortality. The two-proportions z-test shows the mortality rate in asthma patients has a significant difference from mortality rate in patients without CRD unmatched group, with p-value equal to 0.00029.

Table 2 was the corresponding statistical summary table of the case and unmatched control groups. In the case group, there were 5557 (100%) asthma patients. Approximately 27.4% (n=1523) of people were identified as Hispanic or Latino, while in the control group, 27.0% (n=11616) were identified as Hispanic or Latino. In the control group, the percentage of the minority group has increased compared to the case group. White people accounted for the majority in the case group (62.4% (n=3467)) and still accounted for the majority in the control group, but the proportion was small, 58.8% (n=25312). Meanwhile, only 12.5% (n=693) of African Americans were in the case group, 13.2% (n=5701) of African Americans were in the control group. No matter in case group or control group, patients whose BMI is identity as overweight and obese are the majority group (respectively 78.8%, 71.5%). Underweight patients in both case and control groups only take 1.1 % 1.7%, respectively.

The conditional logistic regression results are presented in Table 3 as the odds ratio (OR) of each variable. Asthma patients compared to patients without CRD were significantly less likely (OR=0.7) to be deceased due to COVID-19 (p-value < 0.001). Race variable was as a significant confounder. Compared with Whites, African Americans were 1.88 times more likely to die from COVID-19 (p-value < 0.001). Compared with White, Others were 1.87 times more likely to be deceased from COVID-19 (p-value < 0.001). Hispanic or Latino patients were more likely to be deceased from COVID-19 when compared with patients who were non-Hispanic or Latino (OR=1.44, p-value < 0.023). Two BMI levels (underweight, overweight and obesity) didn’t show a significant association with COVID-19 mortality. Compared with normal weight patients, underweight patients were 1.40 times more likely to die (p-value < 0.363) however this relationship didn’t reach statistically significant level.

## 4. Discussion

The conditional logistic regression results showed that asthma patients had a lower risk of death when compared with non-CRD patients within each stratum. That shown asthma would not increase the risk of death compared with non-CRD patients at a similar age, gender comorbidity score. Previous studies demonstrated that asthma patients with COVID-19 had a high prevalence of comorbidities, including hypertension, heart disease, diabetes, and obesity [6]. Thus, not accounting for comorbidity status could have biased some studies and result in a conclusion that asthma increases mortality risk in COVID-19 patients [2]. Because of the feature selection and propensity matching in our study that included comorbidity score, this potential bias could have been alleviated. That could explain why our study showed that COVID-19 positive asthma patients had a lower death rate.

Propensity-score matching based on gender, comorbidity status, and age status in a case-control study allowed an adjustment for confounding and improved the study design. The imbalance between number of patients with asthma and patients without CRD in the original dataset was reduced by the 1:2 matching procedure. Each patient in the case group was matched with two patients in the control group that attenuated the effect of unmeasured confounders. Overall, there were 5,557 strata. Conditional logistics regression ensured the statistical power of subgroup analysis and interaction tests [7]. The estimated coefficients that were calculated by conditional logistic regression could only be interpreted within each stratum. Patients with other chronic diseases like diabetes may increase mortality rate. African Americans had a higher risk of death when tested positive for COVID-19 compared with Whites. Hispanic or Latino patients had also a higher mortality rate. BMI levels (overweight and underweight) did not reach a sufficient level of significance as a mortality risk factor in COVID-19 patients. Our study demonstrated the power of a nation-wide big data resource and utility of a secure data enclave for conducting big data analyses using case-control design.

## 5. Conclusion

When analyzing the COVID-19 mortality, underlying chronic respiratory conditions may play an essential role. Covid-19 positive asthma patients had a lower death rate than non-CRD patients. Using propensity-score matching in case-control design, we reduced the confounding effect of gender, age, and comorbidity with the. Race and ethnicity were important factors in the analysis of COVID-19 deaths.

## Figures and Tables

**Table 1.** Mortality Rate of patients with asthma and without CRDs.

	Death due to COVID	Alive	total	% Death Rate
With Asthma	159	5398	5557	2.9%
Without CRD (In Unmatched group)	1658	41398	43056	3.9%

**Table 2.** Descriptive statistics of variables in case group and control group

		Patients only has asthma (Case) N=5557		Patients without CRD (control) in unmatched group N= 43083	
Variables Name		Numbers of patients	%	Numbers of patients	%
Ethnicity	Hispanic or Latino	1523	27.4%	11616	27.0%
	Not Hispanic or Latino	4034	72.6%	31467	73.0%
Race	White	3467	62.4%	25312	58.8%
	Black or African	693	12.5%	5701	13.2%
	Others	1397	25.1%	12070	28.0%
BMI	Underweight	62	1.1%	741	1.7%
	Normal weight	1115	20.1%	11546	26.8%
	Overweight and obesity	4380	78.8%	30796	71.5%
Asthma	Has Asthma	5557	100.0%	0	0.0%
Age status	18–35	1632	29.4%	12178	28.3%
	36–50	1422	25.6%	10401	24.1%
	51–75	2124	38.2%	16435	38.1%
	76+	379	6.8%	4069	9.4%
Comorbidity status	0–2	4086	73.5%	34376	79.8%
	3–4	967	17.4%	6652	15.4%
	5–6	321	5.8%	1356	3.1%
	7+	183	3.3%	699	1.6%
Gender (match)	MALE	1837	33.1%	20100	46.7%
	FEMALE	3720	66.9%	22983	53.3%

**Table 3.** Result of Conditional Logistic Regression to Identify Mortality Risk Factor Asthma

		Odds ratio	CI (Lower)	CI (Upper)	Pr(>|z|)
Patient	Do not have any CRD	1(ref)	_	_	_
	Asthma	0.7	0.58	0.86	0.001
Ethnicity	Not Hispanic or Latino	1(ref)	_	_	_
	Hispanic or Latino	1.44	1.05	1.96	0.023
Race	White	1(ref)	_	_	_
	Black or African American	1.88	1.39	2.55	< 0.001
	Others	1.87	1.37	2.55	< 0.001
BMI	Normal weight	1(ref)	_	_	_
	Underweight	1.4	0.68	2.9	0.363
	Overweight & obesity	1.01	0.78	1.32	0.912
